# A three-dimensional statistical shape model of the growing mandible

**DOI:** 10.1038/s41598-021-98421-x

**Published:** 2021-09-22

**Authors:** C. Klop, MAGIC Amsterdam, A. G. Becking, C. Klop, J. H. Koolstra, N. H. J. Lobé, T. J. J. Maal, C. S. Mulder, J. W. Nolte, R. Schreurs, V. Vespasiano

**Affiliations:** 1 Department of Oral and Maxillofacial Surgery, Amsterdam UMC (Location AMC) and Academic Centre for Dentistry Amsterdam (ACTA), University of Amsterdam, Amsterdam, The Netherlands; 2 Department of Oral Cell Biology and Functional Anatomy, Academic Centre for Dentistry Amsterdam (ACTA), University of Amsterdam and Vrije Universiteit Amsterdam, Amsterdam, The Netherlands; 3 Department of Radiology and Nuclear Medicine, Amsterdam UMC (Location AMC), University of Amsterdam, Amsterdam, The Netherlands; 4 Department of Oral and Maxillofacial Surgery 3D Lab, Radboud University Medical Centre Nijmegen, Radboud Institute for Health Sciences, Nijmegen, The Netherlands

**Keywords:** Anatomy, Bone, Musculoskeletal development, Computational biology and bioinformatics, Statistical methods

## Abstract

Mandibular growth and morphology are important topics in the field of oral and maxillofacial surgery. For diagnostic and planning purposes, a normative database or statistical shape model of the growing mandible can be of great benefit. A collection of 874 cadaveric children’s mandibles with dental age between 1 and 12 years old were digitized using computed tomography scanning and reconstructed to three-dimensional models. Point correspondence was achieved using iterative closest point and coherent point drift algorithms. Principal component analysis (PCA) was applied to find the main modes of variation in the data set. The average mandible was presented, along with the first ten PCA modes. The first mode explained 78% of the total variance; combining the first ten modes accumulated to 95% of the total variance. The first mode was strongly correlated with age and hence, with natural growth. This is the largest study on three-dimensional mandibular shape and development conducted thus far. The main limitation is that the samples lack information such as gender and cause of death. Clinical application of the model first requires validation with contemporary samples.

## Introduction

The development of the mandible is an important topic in the field of oral and maxillofacial surgery, orthodontics and dentistry, and forensic sciences. Knowledge of normal mandibular growth aids in an early and deliberate diagnosis of developmental disorders with abnormal mandibular growth, such as craniofacial microsomia, micrognathia, macrognathia, unilateral condylar hyperplasia, and other skeletal disharmonies. Normative data would also be appreciated for application in orthodontics, especially for interceptive growth modulation and surgical treatment planning. In forensics, reference data is essential for the purpose of age determination.

The technique of lateral cephalometric radiography has been extensively used to study mandibular growth. By analyzing the altering position of metallic implants, researchers were able to describe the longitudinal growth pattern of the mandible^1–6^. These studies have small sample sizes, which makes it impossible to describe the natural variation in morphology of the mandible. Anatomical reports with larger samples sizes and wider age ranges have contributed to literature by presentation of normative data^7–9^. While cephalometric radiography has proven to be a convenient and inexpensive tool, more advanced imaging techniques can be explored to capture the true three-dimensional (3D) shape of the mandible.

Imaging techniques such as computed tomography (CT), cone-beam CT (CBCT), and, to a lesser extent, magnetic resonance imaging (MRI) have become increasingly popular to study mandibular development. Some studies investigated one aspect of the mandible, such as the condyle^10–13^, ramus^14^, mandibular canal^15–18^, or the lingula^16,19^. Several authors attempted to describe the shape^20–25^ or the growth of the mandible as a whole^26–44^. Limitations of these studies mainly involve a small sample size, narrow age range, selection bias, or extensive manual input, such as landmark annotation. This may lead to an incomplete or inaccurate representation of the mandibular shape. In this study, we present a statistical shape model of the mandible between 1 and 12 years old.

## Methods

### Study samples

A collection of 1083 children’s mandibles were retrieved from the Eastside Cemetery (Oosterbegraafplaats) in Amsterdam, the Netherlands. This cemetery was in use for burial between 1866 and 1894 and was cleared in 1912. The use of the mandibles for research purposes was approved by the Ethical Committee of the Academic Centre of Dentistry in Amsterdam (ACTA) (protocol number 2017012). All methods were carried out in accordance with relevant guidelines and regulations. The dental age of the mandibles was determined, following the method by Schour and Massler^45^. Most samples were estimated to be between 1 and 12 years old; a small number of samples were classified as “older than 12 years”. All physical models were thoroughly inspected and excluded if there was any evident pathology or deterioration present. Mandibles of which the age could not be reliably determined were also excluded, bringing the total sample size ‘fit for study’ to 874. Information on age category and corresponding number of samples can be found in Table 1.

**Table 1:** Sample size per age category.

Age	1	2	3	4	5	6	7	8	9	10	11	12	12+
Samples	12	75	215	187	107	116	47	29	22	28	15	8	13

### Data acquisition

**Figure 1:**
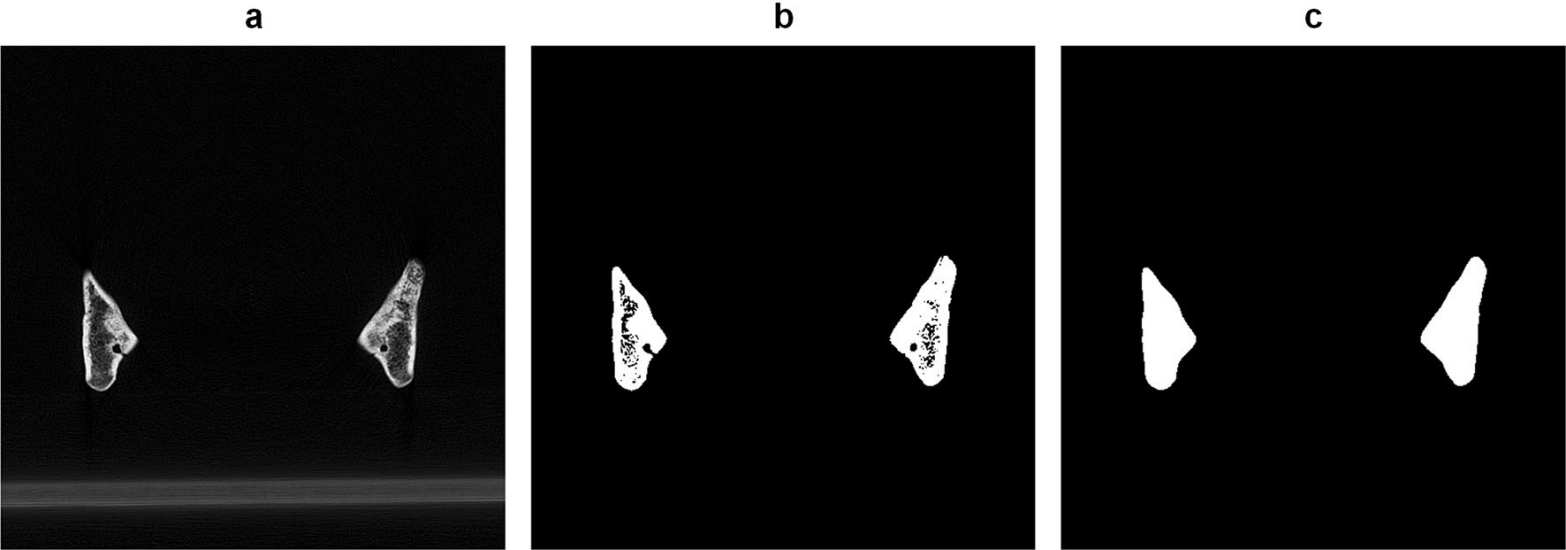
DICOM post-processing. Coronal DICOM slice (**a**), after segmentation (**b**), and after morphological closing and smoothing (**c**).

The mandibles were scanned in a polystyrene template using a Siemens SOMATOM Force multi-detector CT scanner (Siemens Healthineers AG, Erlangen, Germany) following a standardized scanning protocol (100 kV, 175 mA, slice thickness 1.0 mm, pixel spacing 0.2 mm). The CT images were stored as digital imaging and communications in medicine (DICOM) data and imported in MATLAB (version 2019a, MathWorks, Natick, MA, USA). Interpolation to 0.5 mm slice thickness was applied to prevent step artifacts in the 3D reconstruction. Post-processing after interpolation involved a thresholding operation to segment the mandible, a morphological closing operation to fill dental alveoli and the mandibular canal, and a smoothing operation (Fig. 1). A stereolithographic (STL) 3D model was reconstructed from the binary volume. No manual input was required during this process.

**Figure 2:**
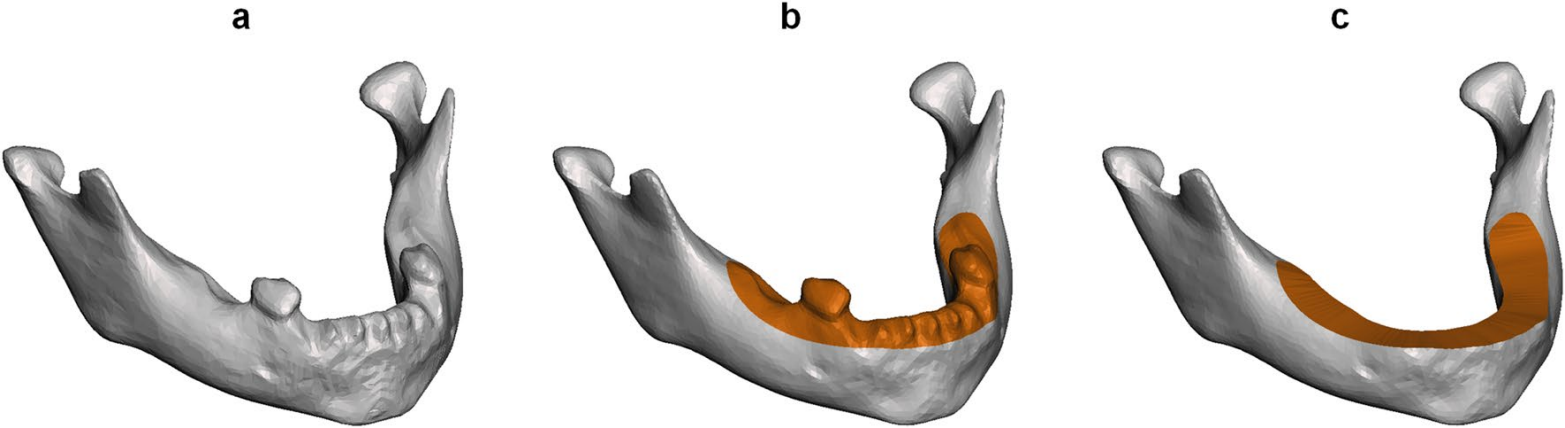
STL post-processing. Original reconstruction (**a**), after remeshing and manual selection (orange region) of the dental region (**b**), and after leveling (**c**).

The 3D models were imported and resampled using the *relative density remesh* function in Meshmixer (version 3.5.474, Autodesk Inc., San Rafael, CA, USA). The purpose of this process was to obtain a homogenous, regular mesh, while retaining a similar number of polygons. The dental region, including erupted teeth and alveolar bone, was manually selected on the 3D model and leveled (flattened) using the *erase and fill* function in Meshmixer (Fig. 2). This was done to minimize the impact of the dental region on the statistical shape model.

### Correspondence mapping

Building a statistical shape model requires correspondence mapping, i.e., consistent sampling of points among all samples. In order to achieve correspondence, a random sample was selected and one side was mirrored over the midsagittal plane to obtain a synthetic, fully symmetrical template with 5801 semi-landmarks (points). In order to minimize the impact of the choice of template, an iterative process was implemented. The template was mapped onto a set of 100 samples using the template-to-target registration as described below, which was fully automated in MATLAB. The average of these 100 mappings was calculated to obtain a new, improved template. The template-to-target registration process was then repeated for each of the 874 samples.

The template-to-target registration comprised the following steps: (1) affine registration with nine degrees of freedom (translation, rotation and non-uniform scaling in three dimensions) using the iterative closest point (ICP) algorithm^46^ and (2) non-rigid registration using the coherent point drift (CPD) algorithm^47,48^. The CPD algorithm involves two smoothing/regularization parameters, which control the coherence of point movement during registration. The non-rigid registration was applied in three stages with decreasing smoothing/regularization parameters to achieve a progressively better fit. The optimal parameter values were determined through a systematic testing approach that evaluated possible combinations in a step-wise fashion.

The resulting 874 mappings were rigidly aligned using the generalized Procrustes algorithm^49^ with six degrees of freedom (translation and rotation in three dimensions). As a result, the scaling difference of the samples was retained. Each mapping was converted into a shape vector of size 17,403 × 1 (number of semi-landmarks, 5801, multiplied by the number of dimensions, 3), thus regarding each coordinate as an independent variable^27^. Concatenating all shape vectors yielded the shape matrix of size 17,403 × 874.

### Principal component analysis

The shape matrix was further analyzed in MATLAB by calculating the mean shape vector and the covariance matrix. The mean shape vector is equivalent to the average mandible, while the covariance matrix contains the linear relationships between all combinations of coordinates. Principal component analysis (PCA) was used to compute the eigenvectors and eigenvalues of the covariance matrix. Each eigenvector describes the principal component (PC) mode of variation in the data set, while the corresponding eigenvalue represents the proportion of total variance that is explained by the eigenvector. PCA assigns a weight to each sample for all PC modes. This PC mode weight is a standard deviation, which represents the dispersion of a sample relative to the mean of the data set. Accordingly, the average mandible has a weight of 0 for all PC modes.

### Statistical analysis

The inter-observer reliability was quantified using the intra-class correlation coefficient (ICC), which was calculated in SPSS (version 26, IBM Corp., Armonk, NY, USA). Relationships between variables were analyzed in MATLAB using Spearman’s rank correlation test. A p-value of less than 0.05 was considered statistically significant. The statistical shape model was evaluated using three commonly used parameters: compactness, generalization ability and specificity^50^. Compactness is the ability of the model to require as few parameters (PC modes) as possible to describe shape instances. A scree plot and Horn’s parallel analysis^51^ with 25 simulations were used to determine the number of dominant PC modes in the model. Generalization evaluates the ability to represent new shape instances, which were not part of the initial model. This was measured through leave-one-out cross-validation: the model was rebuilt using all but one sample, which was then reconstructed and compared to its respective original. This process was repeated for all samples and the average point-to-point differences were documented. Specificity is the ability to represent only valid instances of the object. This was quantified by generating 1000 random, normally distributed instances of the object and documenting the average point-to-point distance to their nearest member in the original model.

### Validation studies

The accuracy of the reconstruction process from CT scan to 3D model was validated by 3D photo scanning. Three randomly selected mandibles were surface scanned using a Scan in a Box 3D optical scanner (Open Technologies Srl, Rezzato, Italy). The photo scans were superimposed on their corresponding 3D reconstruction. Differences between the models were visualized and analyzed using distance maps.

The teeth removal procedure was executed by one of three observers (CK, CSM and VV), but an inter-observer reliability study was carried out first. Fifteen randomly selected samples were processed by all observers and the resulting shapes were compared. The statistical shape model was used to calculate the PC weights of these 3 × 15 samples, which were then compared to find their ICC values.

Two validation steps were implemented in Blender (version 2.92, Blender Foundation, Amsterdam, the Netherlands) to evaluate the geometrical and anatomical correctness of the template-to-target registration procedure. The geometrical correctness was validated by overlaying 100 randomly selected mappings with their respective original 3D model. A ray casting procedure was used to calculate the distance between the two models and the mean distance was documented. The anatomical correctness was validated using the same series of 100 mappings. Seven distinct landmarks were visually compared between the template and the mappings. The following landmarks were used: both lateral condylar processes, both coronoid processes, both mandibular angles, and mental protuberance.

## Results

### Validation studies

The 3D reconstruction process was validated by overlaying three reconstructions with their corresponding 3D photo scan. A deviation of < 0.5 mm was observed on relatively flat surface areas (e.g., mandibular body and ramus) and < 2.0 mm on curved surface areas (e.g., coronoid process).

The inter-observer reliability of the teeth removal procedure was studied by comparing the resulting shapes of 15 randomly selected mandibles. Most PC modes had excellent reliability; only modes 3 and 4 seemed to be influenced by different observers, as is shown in Table 2.

**Table 2:** Intra-class correlation coefficient (ICC) with 95% confidence interval (CI) for the first ten principal components.

Mode	ICC	95% CI
1	0.99	(0.96–1.00)
2	1.00	(0.97–1.00)
3	0.86	(0.50–0.96)
4	0.80	(0.40–0.93)
5	1.00	(0.99–1.00)
6	0.99	(0.95–1.00)
7	1.00	(0.99–1.00)
8	0.99	(0.97–1.00)
9	0.98	(0.94–0.99)
10	0.96	(0.90–0.98)

The template-to-target registration process was validated for geometrical and anatomical correctness. Overlaying 100 mappings with their respective original 3D model was used for geometrical validation, which resulted in a mean difference of 0.05 mm. The anatomical correctness was validated by comparing landmarks between the mappings and the template; a submillimeter difference was seen between the actual and expected landmark positions in the majority of the cases.

### Statistical shape model

An adequate template-to-target mapping result was initially reached in 871/874 (99.7%) cases, using equal smoothing/regularization parameters. These parameters were empirically adjusted to obtain a successful registration in the remaining three cases.

**Figure 3:**
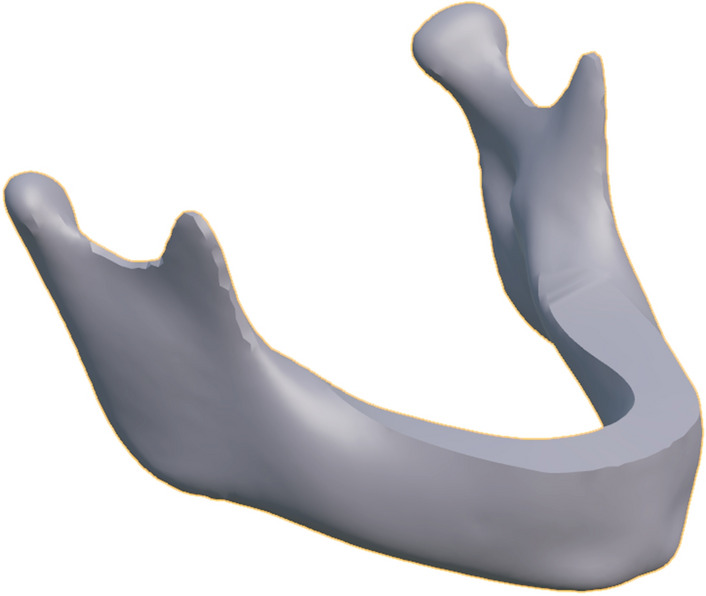
The average mandible.

**Figure 4:**
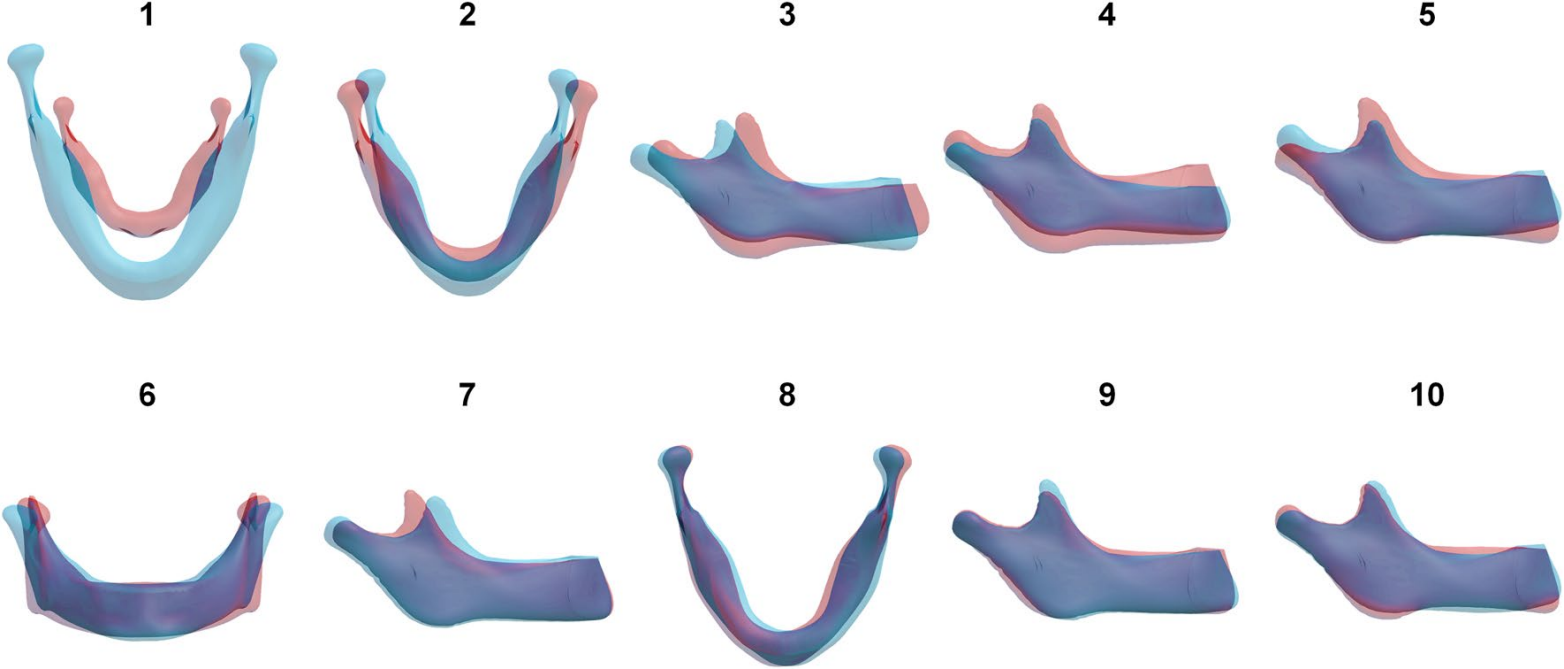
Principal component modes 1–10. For each mode, two models are shown: a weight of − 3 standard deviations (red) and a weight of + 3 standard deviations (light blue). Dark blue zones represent overlap between these models.

**Figure 5:**
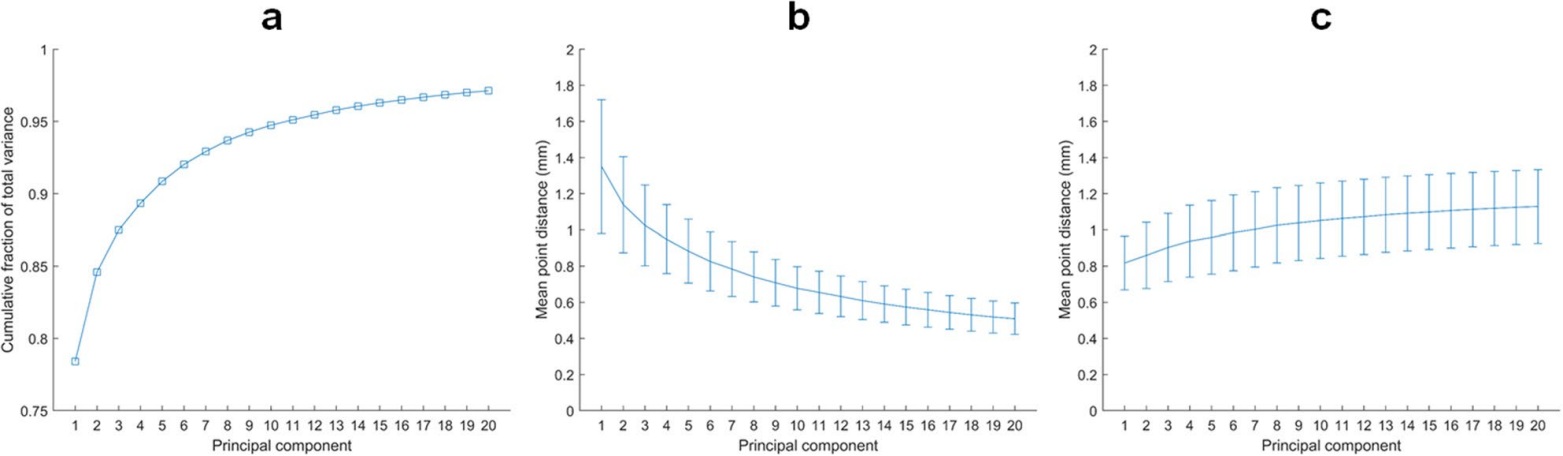
Compactness (**a**), generalization ability (**b**) and specificity (**c**) plots for the first 20 principal components of the statistical shape model. The compactness plot (**a**) shows the cumulative fraction of total variance. The generalization ability plot (**b**) displays the average distance between the original and reconstructed samples using leave-one-out cross-validation. The specificity plot (**c**) shows the average distance between randomly generated instances and their nearest member in the original data set.

PCA was applied to give insight into the average shape and shape variation of the data set. Horn’s parallel analysis revealed that the first 16 modes had an eigenvalue greater than the 95th percentile of the simulated eigenvalues. The first mode alone explained 78.4% of the total variance in the data set; combining the first ten modes accumulated to 94.7% and the first sixteen modes to 96.5% of the total variance. For the sake of brevity, only the first ten modes are considered. The average mandible is shown in Fig. 3 and the first ten PC modes are demonstrated in Fig. 4. The compactness plot, generalization ability, and specificity of the statistical shape model are shown in Fig. 5.

Each PC mode represented one or multiple physical processes; mode 1 corresponded primarily to the overall size of the sample, mode 2 to length/width ratio, mode 3 to wide vs. narrow mandibular angles combined with symphysis height, mode 4 to overall height, and mode 5 to backward vs. forward bending of the ramus and chin. Mode 6 represented a square vs. triangular shape in frontal view, mode 7 coronoid position in anteroposterior direction, mode 8 chin skew to the right vs. to the left, mode 9 coronoid height, and mode 10 a combination of antegonial notch depth and upward vs. downward growth of the chin. Overall increase in size (mode 1) appeared to be non-uniform. Relatively, height (craniocaudal direction) increased more than length (anteroposterior direction), and length increased more than width (mediolateral axis). The first mode comprised more subtle phenomena as well; while overall size increased, the chin advanced and the mandibular angles became more acute. The first ten PC modes are visualized as movie sequences, in which these shape variations can be studied in greater detail (Supplementary Material, videos 1–10).

**Figure 6:**
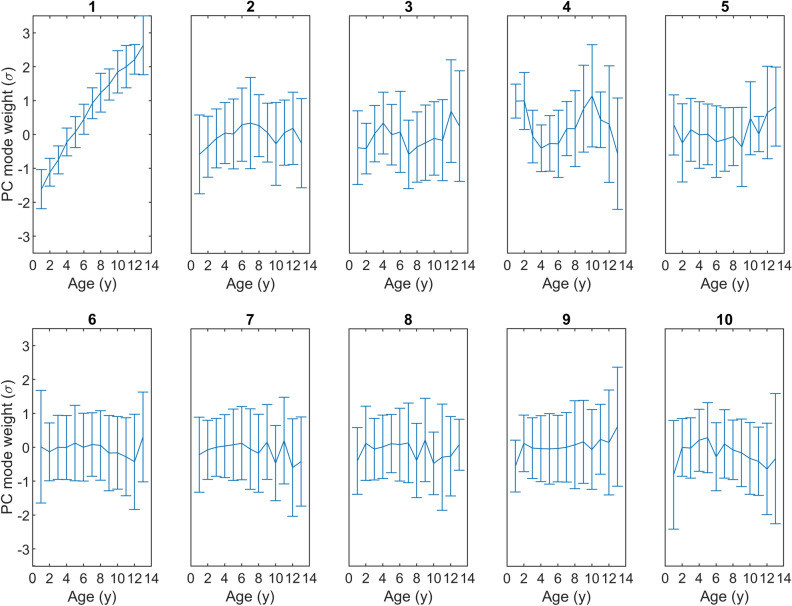
Principal component (PC) weight in standard deviations (vertical axis) plotted against dental age (horizontal axis) for PC modes 1–10.

Relationships between PC mode weight and dental age are shown in Fig. 6. The first mode in particular revealed a strong and significant correlation with age (r_s_ = 0.87, p < 0.001). Other correlations of PC mode weight with age were weak or not significant. PC mode 4 might exhibit a sinusoidal correlation, a relationship that is not exposed by Spearman’s r_s_.

**Figure 7:**
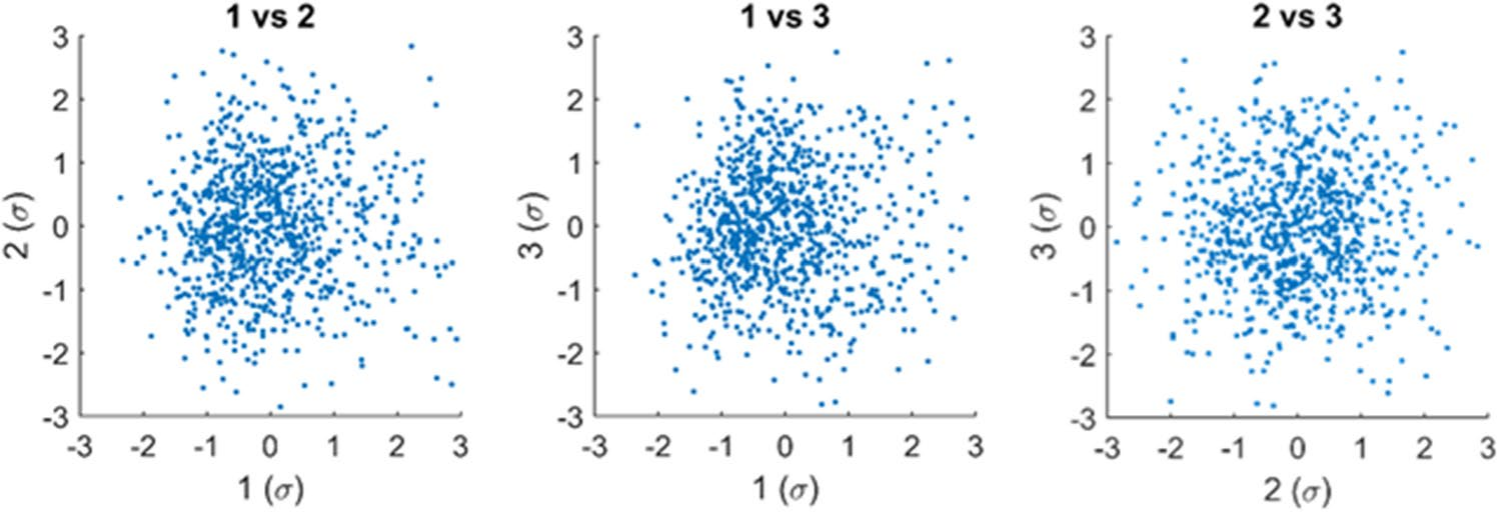
Principal component weights in standard deviations plotted against each other for modes 1–3.

The first ten PC modes were plotted against each other, none of which revealed a relevant dependence or clustering. In Fig. 7, the relationships between the first three PC modes are shown as an example.

## Discussion

The literature on the average three-dimensional shape and shape variation of the mandible is incomplete. Dating back from the second half of the twentieth century, the works of Björk, Skieller, Ødegaard and Baumrind have provided pivotal insights in the development of the mandible using two-dimensional imaging techniques^1–6^. Later publications exploited large longitudinal databases of lateral radiographs (e.g., Fels Longitudinal Study, Bolton-Brush Growth Study) to investigate craniofacial development and natural variation^7–9^. Since the end of the twentieth century, studies focused increasingly on three-dimensional assessment. Several longitudinal CT-based or MRI-based studies have contributed valuable information on the growth pattern of the mandible^27–29,35,37,38,41–44^. However, these studies often have a limited sample size and are therefore less suitable for analyzing normal variation in morphology. Another common limitation is a restricted age range, which impairs the description of morphology as a function of age. Cross-sectional studies that have investigated the shape of the mandible often suffer from the same limitations^26,36,39^. There are several reports on the natural variation in morphology of the mandible, but these pertain to the adult mandible only^20–25^. Two publications by Chung et al. and one by Chuang et al. primarily focus on technical advancements in the framework for developing a statistical shape model and demonstrate its use by presenting a (preliminary) model of the young mandible^30–32^. Two studies by Coquerelle et al. and one by Remy et al. have considerable age ranges and samples sizes^33,34,40^, and have the most comparable data sets to the current study. Of the research with a similar age range as the present study, four studies employ PCA to investigate the morphology of the mandible^27,33–35^.

A statistical shape model is presented in this study, based on 874 samples of the human mandible. This is the largest conducted study on three-dimensional mandibular development. The average shape and variation of the data set were described using PCA. Interpretation of the resulting PC modes is difficult since they are not necessarily linked to a logical entity to humans. Each of the first ten PC modes was associated with one or two physical processes, but also comprised many subtle processes on close inspection. As each subsequent mode explains a decreasing fraction of the total variance in the data set, it becomes increasingly difficult to give a relevant meaning to that mode. Hence, including all PC modes in the model is unnecessary and ineffective. Based on Horn’s parallel analysis, only the first 16 modes should be incorporated in the model. This is supported by the generalization ability and specificity plots, which approximately stabilized after 16 modes. Combining the first 16 modes resulted in a comprehensive model, as they accounted for nearly 97% of the total variance. In this paper, only the first ten modes are considered for the sake of brevity.

The first single PC mode explained already 78% of the total variance, and a strong positive correlation with age was observed. Both are expected findings since size, which increases with age due to allometric growth, was clearly embedded in the first PC mode. The first mode comprised at least two more phenomena: the chin underwent an advancement and the mandibular angles became more acute with age. These findings have also been associated with natural growth in previous reports^30,32,37,40,44^. Growth appears to be disproportional, as height increased more than length, and length increased more than width. A similar conclusion could be drawn from the data in a publication by Kelly et al.^37^. In some publications, scaling differences between the samples were removed by performing the generalized Procrustes alignment with nine degrees of freedom (translation, rotation and scaling in three dimensions)^27,33–35^. This is in contrast with studies that performed alignment with just six degrees of freedom (translation and rotation in three dimensions) and retained the scaling differences^29,36,38,43^. Even though some authors removed uniform scaling as a factor, the first principal component is still associated with growth^27,33–35^. This corroborates the finding in the current study that growth implies much more than merely a uniform increase in size. In this study, the choice was made to retain the scaling factor, because scale is an intrinsic property of the samples (as opposed to translation and rotation). Having the first PC mode represent allometric growth makes it a convenient parameter for evaluating the size of clinical cases and thus, to diagnose growth disorders with greater confidence.

PC modes are by definition orthogonal and uncorrelated. Plotting them against each other could reveal other meaningful relationships, such as a non-linear structure or clustering. In our case, no relevant dependence or clustering was found between PC modes. No statistically significant correlations with age were found for PC modes 2–10. The majority of age-related processes seemed to be incorporated in the first mode. Although not correlated with age, modes 2–10 could be useful for evaluating asymmetrical or disproportional growth disorders of clinical cases.

The use of ex vivo samples allow for employing a very consistent, standardized and reproducible methodology. CT scanning has proven to be a convenient imaging technique for scanning conserved mandibles. Due to the polystyrene template, the samples were surrounded by air, making segmentation straightforward. This is an advantage compared to using clinical (CB)CT scans, in which segmentation of the condylar area is often challenging^30^. Clinical scans often have different scan protocols, depending on the indication for scanning, whereas the scan protocol in this study was equal for all samples. Almost all similar publications also used a polygon mesh for surface representation^27,31,34–42^, because of their benefits in displaying, landmark annotation, and alignment, as opposed to volumetric data. Resampling with a regular mesh was chosen as opposed to an adaptive mesh (where curved surfaces are represented by more polygons than flat areas) to obtain a homogenous point distribution. The disadvantage of using an adaptive mesh is that the CPD and PCA algorithms are dependent on the point sampling density, and would thus assign more weight to curved areas. Using a regular mesh, on the other hand, caused some detail loss on curved areas, such as the coronoid. In most similar papers, study samples were used with an intact dental region, which was incorporated in the segmentation^36–40^. In this study, erupted teeth and alveolar bone were manually selected and leveled, as these would lead to a substantial distortion of the shape model in the dental area. This manual operation took approximately two minutes per sample. A (semi-)automated method could be developed to perform this task. The teeth removal procedure seemed to have influenced PC mode 3 and 4 to some extent, but their ICC values (0.86 and 0.80 respectively) were well within acceptable limits. It is expected that these modes are affected by this procedure, since these modes were related to the height of the dental area. The CPD algorithm was used to establish point correspondence between the samples, with decreasing smoothing/regularization parameters. These parameters were empirically chosen, as there are no clear recommendations for these values except for the ranges that were suggested by the developers of the algorithm^47,48^. Preliminary testing on a small number of samples aided in determining the optimal values. The authors experienced that there is some leeway in these values for obtaining an adequate mapping result of the mandible. The concept of applying the smoothing/regularization parameters in three stages with decreasing values was proposed by the developers of the algorithm^48^. This approach was successful in the vast majority of the cases. In the remaining three cases, different values were chosen to obtain an adequate result. Ultimately, the values of these parameters are only a means to the end of achieving robust and accurate point correspondence. The robustness of the registration procedure was corroborated by the results of the geometrical and anatomical validation studies. There was a negligible difference between the mappings and their original 3D model and anatomical landmarks were well within acceptable margins from their expected positions. By applying the CPD algorithm, manual landmark annotation was circumvented. In contrast, manual landmark annotation was used in multiple similar studies, which is often labor-intensive and may introduce inter-observer and intra-observer variability^26,36–42,44^.

The data set that was used in this study had an uneven age distribution, as most samples were between 2 and 6 years old. The age distribution could have an effect on the PCA, since it is theoretically possible that some morphological aspects exhibit larger variations in younger samples than in older samples, or vice versa. The authors argue that, although this is a potential effect, it does not warrant the exclusion of valuable data to make the age distribution more equal. Caution should be exercised when applying the model in age categories with a higher risk of selection bias. This statement especially pertains to age category 12, with a small sample size of 8, and age category 13, in which all samples older than 12 years were included.

The most important limitation of this paper is that information on internal and external conditions, such as gender, chronological age, comorbidities, facial growth and occlusion patterns, cause of death, and nutritional conditions, are unknown. Measures were taken to exclude samples with evident pathology or deterioration. While these outliers were removed—which would have had the most effect on the model—it cannot be ruled out that some pathological samples were included. This is a comparable limitation in many studies with contemporary, clinical subjects: these cases may not comprise a reliable cross-section of the normal population. In cases where children are subjected to CT examinations, there are often reasons as to why the mandible is unusable for a statistical shape model. This includes pathology such as trauma or congenital diseases, but also a limited field of view due to radiation protection guidelines.

Instead of chronological age, the dental age was estimated and used as an independent variable. Several reports indicate that the Schour and Massler method is fairly accurate for age estimation, especially up to the age of 12 years^52–54^. Some papers report an underestimation of chronological age by this method^52,54^, others a slight overestimation^53^. The absolute mean difference between dental and chronological age is approximately one year^52,54^. Large deviations between dental and chronological age, or differences in morphology between the study samples and the current population, may influence clinical decision-making. Hence, it is essential to validate the model using contemporary samples as the final step before clinical implementation.

## Conclusion

A statistical shape model of the growing mandible was presented, based on 874 samples. The morphology of the mandible as a function of (dental) age between 1 and 12 years old was described. Principal component analysis was applied to investigate the primary modes of shape variation. The first mode explained over 78% of the total variance in the data set and was strongly correlated with age. This mode comprised multiple processes; while overall size increased, the chin advanced and the mandibular angles became more acute. It was concluded that the first mode represented allometric growth. There were 16 dominant modes of variation in the model, which were able to capture nearly 97% of the total variance. The most important limitation of this study is that the samples date back to the second half of the nineteenth century and lack information such as gender and cause of death. The final step before clinical implementation is validation of the presented model using contemporary samples.

## Supplementary Information

Supplementary Legends.

Supplementary Video S1.

Supplementary Video S2.

Supplementary Video S3.

Supplementary Video S4.

Supplementary Video S5.

Supplementary Video S6.

Supplementary Video S7.

Supplementary Video S8.

Supplementary Video S9.

Supplementary Video S10.

## Data Availability

The data sets generated during and/or analyzed during the current study are available from the corresponding author on reasonable request.
