# Supplementary material for: A three-dimensional statistical shape model of the growing mandible
Source: Sci Rep. 2021 Sep 22;11:18843. doi: 10.1038/s41598-021-98421-x (PMC8458295; doi:10.1038/s41598-021-98421-x)
Supplement: Supplementary file 1 — Supplementary Legends. [file 41598_2021_98421_MOESM1_ESM.docx]

**SUPPLEMENTARY INFORMATION LEGENDS**

**Supplementary Information videos 1-10:** Visualization of principal component (PC) modes 1-10. The red model in the start of the video has a weight of -3 standard deviations, the blue model in the end of the video has a weight of +3 standard deviations. The grey model in the middle of the video is the average mandible.
